# Dietary branched-chain amino acids and odds of obesity among immigrant Filipino women: the Filipino women’s diet and health study (FiLWHEL)

**DOI:** 10.1186/s12889-022-12863-0

**Published:** 2022-04-05

**Authors:** Akinkunmi Paul Okekunle, Heejin Lee, Sherlyn Mae P. Provido, Grace H. Chung, Sangmo Hong, Sung Hoon Yu, Chang Beom Lee, Jung Eun Lee

**Affiliations:** 1 Department of Food and Nutrition, College of Human Ecology, Seoul National University, 1 Gwanak-ro, Gwanak-gu, Seoul, 08826 South Korea; 2 Research Institute of Human Ecology, Seoul National University, 1 Gwanak-ro, Gwanak-gu, Seoul, 08826 South Korea; 3 Department of Child Development & Family Studies Seoul National University, 1 Gwanak-ro, Gwanak-gu, Seoul, 08826 South Korea; 4 Division of Endocrinology and Metabolism, Department of Internal Medicine, Hanyang University Guri Hospital, Hanyang University College of Medicine, 153 Gyeongchun-ro, Guri, Guri-si, 11923 South Korea

**Keywords:** Branched-chain amino acids, Obesity, Filipino, Immigrant health, FiLWHEL

## Abstract

**Background:**

The dietary environment promoting adiposity keeps evolving and of interest is the significance of dietary branched-chain amino acids (BCAA). This study assessed the association between dietary BCAA intakes and odds of obesity among immigrant Filipino women in Korea.

**Method:**

We included 423 immigrant Filipino women enrolled in the Filipino Women’s diet and health study in the Republic of Korea. Dietary BCAA intakes were estimated from 24 hour recalls and adjusted for energy intake using the residual method. General obesity was derived from direct anthropometric measurements (height, weight and waist circumference – WC) and defined as body mass index (BMI) ≥25 kg/m^2^ and abdominal obesity as WC ≥80 cm. Odds ratios (OR) and 95% confidence intervals (CI) by tertile distribution of energy-adjusted BCAA intakes were estimated using multivariable logistic regression with a two-sided *P < 0.05*.

**Results:**

Median (interquartile range) for BCAA intakes in g/day were; 7.9 (5.8, 10.3) g/day for total BCAA; 2.0 (1.5, 2.6) g/day for isoleucine, 3.5(2.5, 4.6) g/day for leucine and 2.4 (1.8, 3.1) g/day for valine. Mean BMI and WC were 23.6 ± 3.8 kg/m^2^ and 79.8 ± 9.3 cm, respectively. Also, 30.2% (128) had BMI ≥25 kg/m^2^ and 42.0% (178) had WC ≥80 cm. ORs (95%CIs) of general obesity across tertile distribution of energy-adjusted total BCAA intakes were 1.00, 0.81 (0.47, 1.37) and 0.62 (0.36, 1.07; *P for trend* = 0.08). A similar trend was observed across tertile distribution of energy-adjusted isoleucine, leucine and valine intakes. However, there was a statistically significant inverse association between total BCAA intake and odds of general obesity in a subset of non-smokers; 1.00, 0.68 (0.39, 1.20) and 0.55 (0.31, 0.98; *P for trend* = 0.04).

**Conclusion:**

We found a suggestive inverse association between higher dietary BCAA intake and odds of obesity in this sample of immigrant Filipino women, particularly among non-smokers. Prospective cohort studies among the immigrant population will be necessary to verity these findings.

## Background

Obesity is the excessive accretion of fats in the subcutaneous tissues and internal organs [1]. It is a complex and leading health concern involving the multifaceted interaction of lifestyle, genetic and environmental factors [2–4]. Dietary environment promoting obesity in populations are still evolving, and of recent is the significance of branched-chain amino acids (BCAA). Elevated serum concentrations of BCAA has been established to be associated with obesity risk [5, 6] with little information on the significance of dietary BCAA exposure.

BCAA are vital constituents of metabolism [7, 8] with several functions (including regulating glucose and protein metabolism) in the body [9]. They are primarily derived from diets [9] and accounts for more than half of essential amino acids in the mammalian food supply [10]. They are substrates of energy balance and key transducers in nutrient signalling [11]. Epidemiological reports have revealed the significance of dietary BCAA in obesity events with limited data accounting for population difference(s), dietary exposure and potential interaction with other metabolic risk factors.

Most previous epidemiological reports on dietary BCAA intake and obesity have been conducted in country-specific indigenous populations [12–15]; however, there are limited studies on this subject among immigrant populations. Migration is a growing phenomenon that plays a critical role in nutritional transitions and health outcomes. It is unclear if the association between dietary BCAA and obesity differs in an immigrant population. The hypothesis of the dietary BCAA and obesity odds is yet to be tested among the immigrant population. Similarly, only a few studies have assessed the relationship between dietary BCAA and odds of obesity by subgroup analyses of traditional lifestyle factors including age, smoking, alcohol use and history of diseases. For example, some studies have reported that higher dietary BCAA intake was directly associated with the risk of diabetes mellitus [16, 17]. However, few studies have tested the relationship between dietary BCAA intakes and odds of obesity according to the history of chronic diseases. Understanding the diet-disease relationship in immigrant populations is important for discerning the significance of shifting dietary exposures’ in disease outcomes. Also, whether these differences could be attributed to the diversity of the study population(s) is yet to be clearly understood.

In this study, we explored the association between dietary intake of BCAA (isoleucine, leucine and valine) and odds of obesity (by subgroup analyses of age, smoking, current alcohol use and history of chronic diseases) among a sample of immigrant Filipino women in Korea.

## Methods

### Study population

The Filipino women’s diet and health study (FiLWHEL) is an ongoing study among immigrant Filipino women ≥19 years in Korea. The study started in 2014 and was designed to assess the significance of health-related behaviour(s), lifestyle and acculturation on the progression of cardiovascular diseases among Filipino women in Korea. The Institutional Review Board of Sookmyung Women’s University (SMWU-1311-BR-012) approved the study, and all participants provided written informed consent. Participants were sampled by convenience from several cities and provinces in Korea from 2014 to 2016. A total of 504 Filipino women were recruited into the study. Details of the protocol, recruitment of participants [18] and preliminary observations [19–21] in the FiLWHEL study has been reported elsewhere [18–21].

Briefly, interviewer-administered questionnaires were deployed to collect demographic, health-related behaviour, medical history, quality of life and acculturation information. Also, anthropometric assessment and 24-h recalls were carried out in person. For the 24-h recall, portion sizes were estimated using food miniatures, photographs, household measures, weight/volume, and standard units and portions. Filipino staff fluent in the Filipino language conducted all interviews using the same protocol across all sites under the supervision of the principal investigators. Also, all information on the questionnaire were inspected on-site, questionnaires were checked (to clarify any inconsistencies), and double-checked for data reliability before data entry. Of the 504 women enumerated in the study, 81 were excluded for the following reasons; pregnant and lactating (*n* = 68) or missing information (diet, *n* = 07 and anthropometry, *n* = 06). The final analysis of the report was based on 423 Filipino women with complete information on dietary and anthropometric data.

### Dietary BCAA intake assessment (exposure)

Dietary BCAA; isoleucine, leucine and valine were derived from a one-day 24-h recall. Participants recollected information on all food items, portion sizes or amount consumed in the previous day preceding the survey. Nutrient data (including BCAA and total energy intakes) were computed using the computer-aided analysis program (Can-Pro 4.0, The Korean Nutrition Society, Seoul, Korea) for professionals [22]. Where food information was unavailable, the food composition tables of the Food and Nutrition Research Institute of the Philippines (for Filipino diets) [23], Korean Rural Development Administration [24], US Department of Agriculture [25] or manufacturers’ information were used to derive nutrition information.

### Anthropometric measurements and ascertainment of obesity (outcome)

Anthropometric measures were collected in keeping with standard protocol. Height and waist circumference (WC) were collected to the nearest 0.1 cm (in a standing position without shoes) using a stretch-resistant tape rule. WC was measured at the midpoint between the lowest border of the rib cage and the uppermost lateral border of the right iliac crest. Also, weight (in kg) was measured using bioelectric impedance equipment (In Body 620, Biospace Company Limited, Seoul, Korea). Body mass index (BMI) was estimated as weight (kg) divided by height (m) square. General obesity was defined as BMI ≥ 25 kg/m^2,^ and abdominal obesity was defined as WC ≥80 cm according to cut-offs for populations in the Asia-Pacific region by the World Health Organization (Western Pacific Regional Office), the International Association for the Study of Obesity, and the International Obesity task force [26].

### Demographic and lifestyle characteristics (covariates)

Participants provided information on age (in years), length of stay in Korea (in years), highest education completed (and classified as ‘elementary to high school’ or ‘college education and above’), current employment status (no, yes), ever smoked (no, yes) and current alcohol use (no, yes). The average number of hours and the number of days spent on physical activity (moderate, vigorous or walking) were provided and vigorous physical activity was defined as having spent at least an hour daily of vigorous physical activity. Also, participants were asked if they have been diagnosed with diabetes or hypertension by a certified clinician or are currently taking medications to lower blood glucose or pressure, respectively.

### Statistical analysis

Isoleucine, leucine, valine and total BCAA (the summation of isoleucine, leucine and valine) were adjusted for energy intake using the residual method [27] and categorized into tertiles to include a reasonable number of participants in each category. Characteristics of participants (mean ± SD or n(%)) were presented across tertile distribution of energy-adjusted BCAA intakes. Logistic regression was used to estimate the odds ratio (OR)s and 95% confidence interval (CI)s of obesity odds by tertile distribution of energy-adjusted BCAA intakes. We assessed changes in ORs of exposure when deciding variables to be included in the final model. First, we adjusted for age (in years, continuous). In Model 1, we adjusted for age (in year, continuous), years of stay in Korea (≤4, 5-9, ≥10 years), education (elementary to high school, college education and above), employment (unemployed, employed), ever smoked a cigarette (no, yes), current alcohol use (no, yes) and energy intake (in kcal/d, continuous), Model 2 was additionally adjusted for history of chronic disease such as; diabetes (no, yes) and hypertension (no, yes). Test for trend was carried out by assigning the median value of tertile distribution as a continuous variable in the model. Furthermore, we conducted subgroup analyses to examine if the association between energy-adjusted total BCAA and odds of obesity varied by age (< 35 or ≥ 35 years), ever smoked (no, yes), current alcohol use (no, yes) or self-reported history of diabetes (no, yes) and hypertension (no, yes). Test of interaction was conducted using likelihood ratio test. All statistical analyses were conducted using SAS 9.4 (SAS Institute Inc., Cary, NC, USA) at a two-sided *P* *<* 0.05.

## Results

A total of 295 controls, 128 cases of overweight/obesity (BMI ≥ 25 kg/m^2^) and 178 cases of abdominal obesity (WC ≥80 cm) were identified in this sample. Mean BMI and WC were 23.6 ± 3.8 kg/m^2^ and 79.3 ± 9.3 cm, respectively. The characteristics of the participants by tertile distribution of total BCAA intakes are presented in Table 1. Women in the third tertile of total BCAA intakes were older, presented a higher proportion of participants with a history of diabetes, had higher energy and protein intakes than those in the first tertile of total BCAA intakes. The median values (interquartile range) of total BCAA intakes across increasing tertile distribution of energy-adjusted total BCAA were 4.8 (3.5, 5.8) g/d, 7.9 (7.2, 8.7) g/d and 11.4 (10.3, 13.6) g/d.

**Table 1:** Characteristics of participants by tertile distribution of *total* BCAA intake in the FiLWHEL study

Characteristics	Tertile distribution of energy-adjusted *total* BCAA intake		
	T1	T2	T3
*N*	141	141	141
Age (years)	34.4 ± 8.1	35.7 ± 8.3	35.6 ± 7.4
< 35	74 (52.5)	69 (48.9)	65 (46.1)
≥35	67 (47.5)	72 (51.1)	76 (53.9)
Length of stay (years)	7.8 ± 5.1	7.5 ± 4.6	7.7 ± 4.9
≤4 years	44 (31.2)	41 (29.1)	39 (27.7)
5 – 9 years	49 (34.8)	59 (41.8)	62 (44.0)
≥10 years	48 (34.0)	41 (29.1)	40 (28.3)
Education			
Elementary to high school	44 (31.2)	42 (29.8)	44 (31.2)
College education and above	97 (68.8)	99 (70.2)	97 (68.8)
Employment status (*Yes*)	71 (50.4)	76 (53.9)	84 (59.6)
Ever smoked (*Yes*)	12 (8.5)	9 (6.4)	15 (10.6)
Current alcohol use (*Yes*)	84 (59.6)	83 (58.9)	80 (56.7)
Vigorous physical activity (*Yes*)	31 (22.0)	22 (15.6)	26 (18.4)
History of diabetes^a^ (*Yes*)	2 (1.4)	2 (1.4)	6 (4.3)
History of hypertension^a^ (*Yes*)	11 (7.8)	9 (6.4)	9 (6.4)
BMI (kg/m^2^)	23.7 ± 3.8	23.4 ± 3.6	23.7 ± 4.3
≥ 25 kg/m^2^	48 (34.0)	42 (29.8)	38 (26.9)
WC (cm)	79.4 ± 8.8	78.9 ± 8.8	79.7 ± 10.4
≥ 80 cm	60 (42.6)	63 (44.7)	55 (39.0)
Total energy intake^b^ (*kcal/d*)	1699.2 (1273.0, 2385.7)	1502.0 (1158.7, 1905.4)	1753.9 (1366.6, 2227.2)
Total protein intake^b^ (*g/d*)	60.2 (37.1, 78.6)	59.0 (44.8, 77.9)	77.9 (62.1, 98.5)
Total BCAA intake^b^(*g/d*)	4.8 (3.5, 5.8)	7.9 (7.2, 8.7)	11.4 (10.3, 13.6)

BCAA – branched-chain amino acids adjusted for energy intake using the residual method

Mean ± SD for continuous variables and n(%) for categorical variables

^a^Self-reported clinical diagnosis and/or current use of medication

^b^Median and interquartile range

The multivariable-adjusted ORs (95% CIs) for the general and abdominal obesity across tertile distribution of dietary BCAA intake are presented in Table 2. Total BCAA intake was not statistically significantly associated with the odds of having general obesity, but there was a suggestive inverse association – 1.00, 0.81 (0.47, 1.37) and 0.62 (0.36, 1.07; *P for trend* = 0.08). These findings were similar for isoleucine intake – 1.00, 0.91 (0.53, 1.55) and 0.64 (0.37, 1.07; *P for trend* = 0.09), leucine intake – 1.00, 0.67 (0.39, 1.16) and 0.63 (0.37, 1.07; *P for trend* = 0.09), and valine intake – 1.00, 0.95 (0.55, 1.61) and 0.73 (0.43, 1.25; *P for trend* = 0.25). Similarly, higher dietary BCAA intake was not associated with the odds of having abdominal obesity across tertile distribution of BCAA intakes; total BCAA – 1.00, 1.00 (0.61, 1.66) and 0.76 (0.46, 1.26; *P for trend* = 0.29); isoleucine – 1.00, 1.00 (0.60, 1.66) and 0.74 (0.45, 1.23; *P for trend* = 0.24); leucine – 1.00, 0.68 (0.41, 1.14) and 0.69 (0.41, 1.13; *P for trend* = 0.15); and valine – 1.00, 1.16 (0.70, 1.92) and 0.88 (0.53, 1.46 *P for trend* = 0.60).

**Table 2:** Odds ratios and 95% confidence interval for odds of general and abdominal obesity according to the tertile distribution of energy-adjusted BCAA intakes

	General obesity (BMI ≥ 25 kg/m^2^)				Abdominal obesity (WC ≥80 cm)			
	T1	T2	T3	*P for trend*	T1	T2	T3	*P for trend*
***total *BCAA**								
cases/total	48/141	42/141	38/141		60/141	63/141	55/141	
Median (IQR) intake (*g/d*)^a^	4.8 (3.5, 5.8)	7.9 (7.2, 8.7)	11.4 (10.3, 13.6)		4.8 (3.5, 5.8)	7.9 (7.2, 8.7)	11.4 (10.3, 13.6)	
Age – adjusted	1.00	0.78 (0.47, 1.30)	0.68 (0.41, 1.14)	0.15	1.00	1.01 (0.62, 1.64)	0.80 (0.49, 1.30)	0.37
Model 1	1.00	0.80 (0.47, 1.35)	0.63 (0.37, 1.08)	0.09	1.00	1.00 (0.60, 1.65)	0.79 (0.48, 1.30)	0.34
Model 2	1.00	0.81 (0.47, 1.37)	0.62 (0.36, 1.07)	0.08	1.00	1.00 (0.61, 1.66)	0.76 (0.46, 1.26)	0.29
**Isoleucine**								
cases/total	47/141	44/141	37/141		61/141	63/141	54/141	
Median (IQR) intake (*g/d*)^a^	1.1 (0.8, 1.5)	2.0 (1.8, 2.2)	2.9 (2.6, 3.5)		1.1 (0.8, 1.5)	2.0 (1.8, 2.2)	2.9 (2.6, 3.5)	
Age – adjusted	1.00	0.88 (0.53, 1.45)	0.69 (0.41, 1.15)	0.16	1.00	1.01 (0.62, 1.63)	0.77 (0.47, 1.25)	0.29
Model 1	1.00	0.91 (0.53, 1.54)	0.64 (0.37, 1.10)	0.10	1.00	1.01 (0.61, 1.66)	0.76 (0.46, 1.25)	0.28
Model 2	1.00	0.91 (0.53, 1.55)	0.64 (0.37, 1.09)	0.10	1.00	1.00 (0.60, 1.66)	0.74 (0.45, 1.23)	0.24
**Leucine**								
cases/total	50/141	39/141	39/141		65/141	57/141	56/141	
Median (IQR) intake (*g/d*)^a^	2.1 (1.5, 2.5)	3.5 (3.2, 3.9)	5.4 (4.6, 5.9)		2.1 (1.5, 2.5)	3.5 (3.2, 3.9)	5.4 (4.6, 5.9)	
Age – adjusted	1.00	0.66 (0.40, 1.11)	0.67 (0.40, 1.11)	0.12	1.00	0.73 (0.45, 1.19)	0.71 (0.44, 1.16)	0.18
Model 1	1.00	0.66 (0.39, 1.14)	0.64 (0.38, 1.08)	0.10	1.00	0.68 (0.41, 1.14)	0.71 (0.43, 1.16)	0.19
Model 2	1.00	0.67 (0.39, 1.16)	0.63 (0.37, 1.07)	0.09	1.00	0.68 (0.41, 1.14)	0.69 (0.41, 1.13)	0.15
**Valine**								
cases/total	45/141	44/141	39/141		57/141	65/141	56/141	
Median (IQR) intake (*g/d*)^a^	1.4 (1.0, 1.8)	2.4 (2.2, 2.6)	3.4 (3.1, 3.9)		1.4 (1.0, 1.8)	2.4 (2.2, 2.6)	3.4 (3.1, 3.9)	
Age – adjusted	1.00	0.91 (0.55, 1.52)	0.78 (0.46, 1.31)	0.35	1.00	1.15 (0.71, 1.87)	0.90 (0.55, 1.48)	0.68
Model 1	1.00	0.94 (0.55, 1.60)	0.74 (0.43, 1.26)	0.27	1.00	1.16 (0.70, 1.92)	0.91 (0.55, 1.49)	0.68
Model 2	1.00	0.95 (0.55, 1.61)	0.73 (0.43, 1.25)	0.25	1.00	1.16 (0.70, 1.92)	0.88 (0.53, 1.46)	0.60

^a^Branched-chain amino acids were adjusted for energy intake using the residual method; *IQR* Interquartile range; Model 1 was adjusted for age (in years, continuous), year of stay (≤4years, 5-9years, ≥10years), education (elementary to high school, college education and above), employment (no, yes), ever smoke (no, yes), current alcohol use (no, yes) and energy intake (in kcal/d, continuous). Model 2 was adjusted for history of diabetes (no, yes) and hypertension (no, yes) in addition to variables in model 1

The associations between dietary BCAA intakes and odds of general obesity (Table 3) did not vary by age or current alcohol use (*P for Interaction* = 0.87 and 0.95, respectively). We found a similar association when we limited the analysis to participants without a history of diabetes or hypertension. However, higher energy-adjusted total BCAA intake was associated with lower odds of obesity in a subset of non-smoking Filipino women only; ORs (95%CI) were 1.00, 0.68 (0.39, 1.20) and 0.55 (0.31, 0.98; *P for trend* = 0.04). The associations between dietary BCAA intakes and odds of abdominal obesity were different by smoking status (*P for Interaction* = 0.04), but not by age or current alcohol use (*P for Interaction* = 0.30 and 0.35, respectively) (Table 4). Although the association between dietary BCAA intake and abdominal obesity was not statistically significant, there was a suggestive inverse association in a subset of non-smoking Filipino women; ORs (95% CIs) were 1.00, 0.85 (0.50, 1.43) and 0.64 (0.38, 1.09; *P for trend* = 0.10).

**Table 3:** Subgroup analysis of the association between energy-adjusted *total* BCAA intake and odds of general obesity

		Tertile distribution of energy-adjusted *total* BCAA intake^a^				
		T1	T2	T3	*P for trend*	*P for Interaction*
Age						
< 35years	cases/total	21/74	16/69	13/65		
	OR (CI)	1.00	0.94 (0.42, 2.09)	0.66 (0.29, 1.51)	0.33	0.87
≥ 35years	cases/total	27/67	26/72	25/76		
	OR (CI)	1.00	0.73 (0.35, 1.51)	0.57 (0.27, 1.19)	0.13	
Ever smoked						
No	cases/total	44/129	36/132	30/126		
	OR (CI)	1.00	0.68 (0.39, 1.20)	0.55 (0.31, 0.98)	0.04	0.19
Yes	cases/total	4/12	6/9	8/115		
	OR (CI)	1.00	2.32 (0.14, 38.57)	3.36 (0.24, 46.97)	0.37	
Current alcohol use						
No	cases/total	21/57	12/58	17/61		
	OR (CI)	1.00	0.35 (0.13, 0.89)	0.61 (0.26, 1.42)	0.31	0.95
Yes	cases/total	27/84	30/83	21/80		
	OR (CI)	1.00	1.19 (0.61, 2.53)	0.57 (0.27, 1.19)	0.14	
History of diabetes						
No	cases/total	47/139	41/139	35/135		
	OR (CI)	1.00	0.80 (0.47, 1.36)	0.61 (0.36, 1.06)	0.08	N/A^c^
Yes	cases/total	1/2	1/2	3/6		
	OR (CI)^b^	–	–	–		
History of hypertension						
No	cases/total	42/130	40/132	33/132		
	OR (CI)	1.00	0.90 (0.52, 1.57)	0.62 (0.35, 1.10)	0.10	N/A^c^
Yes	cases/total	6/11	2/9	5/9		
	OR (CI)^b^	–	–	–		

*BCAA:* Branched-chain amino acids were adjusted for energy intake using the residual method;

^a^Model was adjusted for age (in years, continuous), year of stay (≤4years, 5-9years, ≥10years), education (elementary and high school, college education and above), employment (no, yes), ever smoked (no, yes), current alcohol use (no, yes), energy intake (in kcal/d, continuous), history of diabetes (no, yes) and hypertension (no, yes);

^b^Insufficient sample

^c^Not available

**Table 4:** Subgroup analysis of the association between energy-adjusted *total* BCAA intake and odds of abdominal obesity

		Tertile distribution of energy-adjusted *total* BCAA intake				
		T1	T2	T3	*P for trend*	*P for Interaction*
Age						
< 35years	cases/total	21/74	29/69	19/65		
	OR (CI)	1.00	1.82 (0.87, 3.81)	0.93 (0.42, 2.02)	0.86	0.30
≥ 35years	cases/total	39/67	34/72	36/76		
	OR (CI)	1.00	0.52 (0.25, 1.07)	0.55 (0.25, 1.13)	0.11	
Ever smoked						
No	cases/total	57/129	57/132	45/126		
	OR (CI)	1.00	0.85 (0.50, 1.43)	0.64 (0.38, 1.09)	0.10	0.04
Yes	cases/total	3/12	6/9	10/15		
	OR (CI)	1.00	4.33 (0.27, 90.56)	8.12 (0.49, 133.08)	0.14	
Alcohol use						
No	cases/total	22/57	22/58	24/61		
	OR (CI)	1.00	0.79 (0.34, 1.83)	0.92 (0.41, 2.07)	0.88	0.35
Yes	cases/total	38/84	41/83	31/80		
	OR (CI)	1.00	1.04 (0.54, 1.98)	0.58 (0.29, 1.14)	0.11	
History of Diabetes						
No	cases/total	59/139	62/139	50/135		
	OR (CI)	1.00	1.00 (0.60, 1.65)	0.72 (0.43, 1.21)	0.21	N/A^c^
Yes	cases/total	1/2	1/1	5/6		
	OR (CI)^b^	–	–	–		
History of hypertension						
No	cases/total	54/130	59/132	49/132		
	OR (CI)	1.00	1.06 (0.63, 1.79)	0.74 (0.44, 1.25)	0.25	N/A^c^
Yes	cases/total	6/11	4/9	6/9		
	OR (CI)^b^	–	–	–		

*BCAA:* Branched-chain amino acids were adjusted for energy intake using the residual method;

^a^Model was adjusted for age (in years, continuous), year of stay (≤4years, 5-9years, ≥10years), education (elementary and high school, college education and above), employment (no, yes), ever smoked (no, yes), current alcohol use (no, yes), energy intake (in kcal/d, continuous), history of diabetes (no, yes) and hypertension (no, yes);

^b^Insufficient sample

^c^Not available

## Discussion

In this study, we found an inverse relationship between higher dietary BCAA intake and odds of obesity among non-smokers, but a suggestive inverse association in the overall sample of this study. Our findings add to the body of literature on the interaction of smoking status in the relationship of dietary BCAA intake and adiposity, accounting for evidence in the context of populations likely experiencing changes in diet pattern and eating behaviour due to migration.

The significance of dietary BCAA intake and odds of adiposity has been tested in some epidemiological reports [12–15, 28, 29], but not in a sample of a population experiencing a change in diet patterns [30–32]. For example, higher dietary BCAA intakes were associated with lower odds of obesity in the INTERMAP population-based survey from China [15]. Similarly, higher dietary BCAA intake was inversely related to the prevalence of overweight and adiposity-related metabolites independent of genetic differences in another population-based survey from the United Kingdom [12]. Because BCAA are derived from diets, the overall dietary exposure should be considered in the relationship of dietary BCAA with obesity. In tandem with this observation, some reports have established that the relationship between dietary BCAA intake and diabetes mellitus was primarily within the context of dietary patterns [16, 33]. To date, few studies have tested the interaction effect of history of disease on the relationship between dietary BCAA intake and obesity. Our study did not have a sufficient number of participants with a history of diabetes or hypertension in subgroup analyses. A meta-analysis has demonstrated that the odds of dietary BCAA relationship differ with obesity and diabetes mellitus. In that report, higher dietary BCAA intake was associated with lower and higher odds of obesity and diabetes mellitus, respectively [34].

There are several plausible mechanisms for the inverse association between higher dietary BCAA intakes and odds of obesity. First, some intervention trials have observed a modest reduction in body weight and fat after BCAA supplementation in a clinical trial [35, 36]. BCAA intake may have impacted body weight through the down-regulation of lipogenic factors and improved insulin sensitivities. Poor insulin sensitivities have been linked with obesity, but BCAA intake/supplementation has been linked with improved insulin sensitivities [12], maintenance of lean body [37] and in some cases, modest weight loss [36]. Also, animal trials have demonstrated that increased dietary BCAA improved glucose and lipid metabolism [38, 39] by upregulating the expression of peroxisome proliferator-activated receptor-alpha to prevent diet-induced obesity in mice models [39]. Second, higher BCAA intake may likely promote lower body weight by increasing circulating leptin concentration to reduce appetite and regulate energy metabolism. Rats fed with leucine-enriched diet were found to have 40% decrease in leptin levels [40]. Exogenous leptin administration has been reported to down-regulate appetite, and consequently decrease body weight in animal models [41].

Furthermore, levels of dietary BCAA intake in this sample of immigrant Filipino women were lower than the indigenous Korean population [42, 43] and other populations [17, 33, 44]. There are currently no local studies among indigenous Filipinos in the Philippines to compare our findings. Our study has several strengths; it is the first epidemiological report on dietary BCAA and obesity among an immigrant population likely to be experiencing changes in diet pattern and eating behaviour. Our report observed a potential interaction of smoking status in the relationship between dietary BCAA intake and obesity. The multivariate adjustment for potential confounding alludes to the reliability of our findings. However, a causal relationship cannot be inferred because of the cross-sectional nature of the study design. The generalizability of our findings is likely to be limited, given that convenient sampling was adopted for participant recruitment. Dietary information using 24-h dietary recall in a single time point may not represent overall dietary exposure, and residual confounding or unmeasured factors are potential factors to consider in examining our results. Longitudinal studies from diverse ethnic backgrounds are necessary to clarify the association of dietary BCAA intake with obesity. Future studies should consider the moderating effects of lifestyle factors and disease history to broaden the scientific understanding of this subject. Also, discerning the contributions of the whole spectrum of dietary exposure could fill gaps on the significance of dietary BCAA in the aetiology of obesity and disease outcomes.

## Conclusions

Higher dietary BCAA intakes appeared to exert a suggestive inverse association with odds of obesity in this sample of immigrant Filipino women, and smoking status may modify the observed inverse relationship.

## Data Availability

The data for this study cannot be made publicly available because FiLWHEL is an ongoing study, and during the signing of consent, the participants were not informed that their information would be stored in a publicly accessible database. However, other researchers are welcome to collaborate with the study team under approval procedures posted on the study website (www.filwhel.org). Requests to access the data may be sent to the data access committee (nutepid@gmail.com). Professors Jung Eun Lee (jungelee@snu.ac.kr) and Chang Beom Lee (lekang@hanyang.ac.kr) have access to the entire dataset.

## Abbreviations

BCAA: Branched-chain amino acids
OR: Odds ratio
CI: Confidence interval
WC: Waist circumference
BMI: Body mass index
